# Association of education background with clinical pharmacists’ clinical pharmacy workload in tertiary hospitals of China

**DOI:** 10.1186/s12909-022-03859-w

**Published:** 2022-11-09

**Authors:** Qingran Sun, Lei Chen, Yuankai Huang, Xiaoyu Xi

**Affiliations:** National Medical Products Administration Key Laboratory for Drug Regulatory Innovation and Evaluation, No. 639, Longmian Avenue, Jiangning District, Nanjing, 211198 Jiangsu Province China

**Keywords:** Education background, Workload, Clinical pharmacist, China

## Abstract

**Background:**

Workload of healthcare providers may affect the quality of healthcare. Practical evidences have indicated that healthcare providers are differentially associated with workload due to their different education and training background. Clinical pharmacists are an indispensable part of medical teams. Under the precondition that clinical pharmacists in China generally undertake uneven clinical pharmacy workload, the relationship of workload and clinical pharmacists’ different education backgrounds remains unclear. This study aimed to assess the association between the education background of clinical pharmacists and their clinical pharmacy workload in China.

**Methods:**

A field questionnaire survey using a stratified sampling was conducted to gather data on education background and clinical pharmacy workload through a self-developed instrument. Ordinary least squares regression was used to evaluate the association of the participants’ education background with their clinical pharmacy workload.

**Results:**

A total of 625 clinical pharmacists from 311 tertiary hospitals in China participated. Two levels of education: less than bachelor’s degree in general pharmacy, or doctoral degree in clinical pharmacy was associated significantly with clinical pharmacy workload of the participants. Participants who had credentials of national level and provincial-level specialized training for clinical pharmacists had undertaken more work than those without. Moreover, the participants’ specialized field, such as respiratory medicine and nephrology, was associated with their clinical pharmacy workload.

**Conclusion:**

Enhancing several aspects of education or training among clinical pharmacists in tertiary hospitals in China may help improve their capability to provide clinical pharmacy services. Efforts are needed to improve the education and training system of clinical pharmacists in China.

**Supplementary Information:**

The online version contains supplementary material available at 10.1186/s12909-022-03859-w.

## Introduction

The workload of medical professionals is closely related to healthcare outcomes [1]. By affecting the quality of work and life of healthcare providers, high workload causes occupational stress [2], and eventually influences the quality of healthcare [3]. Clinical pharmacists are an indispensable part of medical teams [4], and their responsibilities include participating in clinical treatment, recording medication-related documents, teaching, carrying out scientific research, and improving their own skills, etc. [5] The clinical pharmacists’ clinical workload is reflected in the coverage rate^1^ and scope of clinical responsibilities [6].

Pharmacy education will expand the knowledge and skills of clinical pharmacists [7–9] to further affect their competency [10, 11]. Such a competency-oriented training model contributes to improve workload of healthcare providers [12, 13]. Therefore, education background may be a potential factor affecting the clinical pharmacists’ workload. Since the contents of pharmaceutical care are different among countries [13], the development of pharmaceutical care may require corresponding specific education and knowledge in different countries. In developed countries, consultation-based prescribing capabilities improve pharmacists’ efficiency [14, 15]. In developing countries, hospital pharmacists rely on drug-centered knowledge [16], while those having received more education carry out clinical work in a wider range [17].

The lag of pharmacy education will restrict the overall development of clinical pharmacy [18]. Clinical pharmacists in China must have bachelor degree or higher qualification in pharmacy or clinical pharmacy and standardized training experience [19]. The clinical pharmacist workforce in China has included professionals with multiple educational levels(from lower than bachelor’s degree to doctoral degree) and diverse professional backgrounds(pharmacy, clinical pharmacy, medicine, pharmacy-related specialties such as pharmacology, nursing and medical English, other specialties not related to pharmacy) [20]. Therefore, it would be helpful to identify the workload differences of clinical pharmacists with different education backgrounds to explore the training direction of clinical pharmacists in China.

The regulations related to the responsibilities and requirements of clinical pharmacists are not explicit in China. For one thing, the absence of clear directions of clinical pharmacists’ responsibilities cause their work being highly arbitrary [21]. Some clinical pharmacists face the dilemma of heavy workload of dispensing [22–24] and lack of the capabilities to participate in clinical treatment [25, 26]. For another, the development of clinical pharmaceutical care projects are generally uneven and imperfect in China [6], where clinical pharmacists’ work still needs to be standardized. Hospital pharmacists undertake complex tasks such as administration and dispensing of drugs, clinical drug treatment, and patient education regarding drug therapy in China. In recent years, the working mode of hospital pharmacists in China has been transforming from drug administration to pharmaceutical care [27, 28]. Developing high-quality pharmaceutical care and training qualified pharmaceutical professionals are the topics of concern in the field of pharmacy education in China [29]. Since the formal education of clinical pharmacy started later in China, yet the school system has not been unified [18, 30]. At present, there is only a national standard for undergraduate clinical pharmacy education (5 years, Bachelor of Science) [31]. The academic degrees, courses, skills and practical requirements of pharmacy, medical and clinical pharmacy are different, so the knowledge and skills acquired by graduates of the above majors vary greatly. The clinical pharmacists in China have not been qualified to undertake the responsibilities related to clinical pharmacy [32–34].

Study has indicated that education and training affect the quality of clinical pharmaceutical care provision [35] whose evidence, however, has been outdated. Besides, there has been research proved that the scope of clinical pharmaceutical care is associated with education background of the director of pharmacy department [36], however, the studied population of this study is not clinical pharmacists. Another study has concluded that as for pharmacists, clinical pharmacy education is an important motivation of provision of pharmaceutical care [37], which just stayed on the oretical discussions. There has been no research to explore the relationship between education background of clinical pharmacists and their clinical pharmacy workload in China.

This study aimed to assess the association between the education background of clinical pharmacists and their clinical pharmacy workload through an empirical survey in tertiary hospitals of China. The findings would act as the reference for the education department and medical colleges in the training direction and curriculum design of Chinese clinical pharmacists. This study may also be valuable to other developing or undeveloped countries.

## Method

### Study design and participants

The healthcare system in China follows a 3-tier hierarchical structure. This study only considered clinical pharmacists from tertiary hospitals because clinical pharmacy services are undeveloped in most primary and secondary health care institutions [38, 39].

The inclusion criteria were as follows: (1) working as full-time clinical pharmacists of the sampled hospitals; (2) undertaking specific duties involving management of pharmaceuticals, patients, or medical information; (3) being available to participate in the study by completing the questionnaire that would take approximately 15–30 minutes; and (4) being willing to sign the informed consent document. Clinical pharmacists in training (students on clerkships or internships) and visiting clinical pharmacists were excluded.

A stratified sampling strategy was adopted. First, all 31 provincial administrative regions (including provinces, autonomous regions, and municipalities) in mainland China were covered in the sampling. Next, cities in each provincial administrative regions were evenly divided into 3 groups according to their 2018 per capita gross domestic product, thereby generating 93 groups. Within each group, 1 city or district was selected using the random number method; thus, 93 cities or districts were selected. In each selected city or district, 2 to 4 tertiary hospitals were surveyed by convenience based on the hospital administrators’ permission. In each surveyed hospital, 2 participants were recommended by the hospital administrator(s) or another participant who completed the survey [40]. Overall, 744 questionnaires were distributed.

### Instrument

An expert panel of 2 administrators and 2 teaching clinical pharmacists from tertiary hospitals, together with 3 experts in clinical pharmacy education from universities, were consulted for the design of the questionnaire. The questionnaire comprised the following 3 sections:

### Covariate

Socio-demographics (gender, age, marital status), technical title, years of practice, specialized field, and features of hospital were included.

### Education background

The clinical pharmacists’ training system in China consists of three parts: medical and pharmaceutical education with record of formal schooling, bases training, individualized training courses provided by training or academic organizations [41]. Therefore, this study identified pharmacists’ education background through three following indicators: higher education background, qualification of practice and training experience.

For higher education background, this section gathered information on whether respondents have obtained a degree of a certain educational level, and the type of degree. Considering that the types of degree of clinical pharmacists are diverse [20], this question was set as a fill-in-the-blank question according to experts’ suggestions. The researchers divided the types of degree into seven groups based on the expert panel’s recommendations, namely (general)pharmacy, clinical pharmacy, Chinese materia medica, (except for the above three) other pharmacy, clinical medicine, (except for clinical medicine) other medical specialties, (except for the above six) other specialties. The qualifications of practice include national-level and province-level standardized training in specialized and general practice. The training experience include certificate of completion of clinical pharmacist training from the Ministry of Health, advanced training certificate of clinical pharmacist, certificate of training faculty of clinical pharmacist of National Health and Family Planning Commission, any overseas training for clinical pharmacist, and (except for the above) other trainings.

### Workload

There was no existing suitable instrument for measuring the clinical pharmacists’ workload in tertiary hospitals in China [42, 43]. This study measured this according to the extent of their clinical work developed. The *Standards of Practice for Clinical Pharmacists (SPCP)* was originally in English and translated by a native speaker of English who is fluent in Chinese and a Chinese translator proficient in Chinese-English translation separately. Subsequently, the 2 versions were reviewed, synthesized, and revised by the 2 translators and the expert panel until they reached consensus on all translations. The final scale was formed with reference to SPCP and *Provisions on the Administration of Pharmaceutical Affairs of Medical Institutions.* A total of 12 questions which covered mainly routine tasks of clinical pharmacists in tertiary hospitals in China were used to measure the extent of clinical work developed of clinical pharmacists. Each question was provided in the form of its complete description and presented to participants as, “Have you ever undertaken the task?” with a response of a 5-level Likert scale, ranging from 1 (strongly disagree) to 5 (strongly agree). It was the total score that was included in regression analysis.

### Pretest

A pretest of the questionnaire was conducted among 47 clinical pharmacists from 24 tertiary hospitals in 6 cities of Jiangsu province in China during April 2019 by convenience sampling. Reliability of the instrument for workload was acceptable (Cronbach ‘s alpha 0.63 for workload instrument). The final questionnaire is available online as Additional file 1: Appendix 1.

### Data collection

A total of 46 undergraduate students majoring in general pharmacy or clinical pharmacy were recruited as data collectors. They were trained to be able to access the potential participant and be familiar with the procedure of conducting the survey and the standardized explanations for potential questions from the participants. Every 2 data collectors investigated 1 set of geographically neighboring cities or districts in pairs during July and August of 2019. After obtaining the hospital administrators’ consent, the data collectors asked the potential participants for their basic information to determine whether they meet the study inclusion criteria. Then they informed the eligible participants of the purposes, contents, and requirements of the survey and confirmed their willingness to participate again. Those who were willing to participate signed the consent form and decided the time and an undisturbed place for survey with the data collectors.

The data collectors orally interviewed the participants with each item of the questionnaire and recorded their responses through an online survey system on mobile phones or tablet computers. Which would convert the data into electronic documents. The data collectors were not allowed to provide any view on the questionnaire, except the requirements or instructions of questionnaire filling. The survey system allowed the users to set restrictions on format of responses and ensured the quality of the data. A total of 5 postgraduates were recruited and trained to review the uploaded documents and immediately return those with data entry errors or damaged data, which were corrected through return visits by data collectors when possible [40].

### Data analysis

Descriptive statistics were used to report the characteristics of the sample. Ordinary least squares regression was used to assess the association of each independent variable with clinical pharmacy workload. Multicollinearity was assessed by examining the variance inflation factor (VIF). An independent variable with a VIF value more than 10 means that it has collinearity within the other independent variables and should be removed. VIF was examined again when an independent variable with the highest VIF more than 10 was removed. This was repeated until multicollinearity was not suspected any more. Three levels of statistical significance were set in this study, namely *p* < 0.1, *p* < 0.05, *p* < 0.01 [40].

To evaluate the robustness of the results, job satisfaction of clinical pharmacists was included in the regression model as a covariable. The similarity of the results of both models could support the relative robustness of the final model. Stata 15.0 was used for data analysis.

## Result

Overall, we distributed a total of 744 questionnaires to 311 tertiary medical hospitals, 625 of which were filled out completely (response rate = 84%). The other 119 questionnaires were excluded due to reasons such as not being collected or uploaded to the survey system, incomplete filling, or corrupted data files. The main characteristics of participants are found in Table 1. The mean age of the participants was 35.06(SD = 6.44), and their mean years of practicing as a clinical pharmacist was 9.3(SD = 6.62). Approximately two-thirds of the whole participants were female (65.6%) and most were married (85.1%). The participants’ mean score of the clinical pharmacy workload was 44.33(SD = 4.65). The score of every activity clinical pharmacists performed is available online as Additional file 2: Appendix 2. Most of the participants were with junior technical title (30.6%) or intermediate title (56.0%). Most of the participants were working in general hospitals (75.5%).

**Table 1:** Sociodemographic Data of Clinical Pharmacists (*N* = 625)

Item	***N***(%)
Age (mean, sd.)	35.06 (6.4)
Years of practice (mean, sd.)	9.3 (6.6)
The score of the completion of clinical pharmacy workload (mean, sd.)	44.33 (6.5)
Gender	
Male	215 (34.4)
Female	410 (65.6)
Marital status	
Unmarried	89 (14.2)
Married	532 (85.1)
Other (divorced, window, etc.)	4 (0.6)
Technical titles	
Junior title	191 (30.6)
Intermediate title	350 (56.0)
Deputy senior title	72 (11.5)
Senior title	12 (1.9)
Type of hospital	
General hospital	472 (75.5)
Specialized hospital	33 (5.3)
Traditional Chinese medicine hospital	82 (11.5)
Other	38 (6.1)
Specialized field ^a^	
Anti-infectives	148 (23.7)
Cardiology	102 (16.3)
Respiratory medicine	89 (14.2)
Gastroenterology	83 (13.3)
Nephrology	32 (5.1)
Oncology	61 (9.8)
Organ Transplantation	8 (1.3)
ICU	39 (6.2)
Endocrinology	48 (7.7)
Neurology	43 (6.9)
Other	133 (21.3)

^a^Some clinical pharmacists engage in two or more specialties, so the sum of this item is not 100%

The participants’ education backgrounds are found in Table 2. Most of the participants’ higher education background was bachelor’s (53.4%) or master’s degree (39.5%). Most of the participants with a bachelor’s degree were in majors of pharmacy (47.4%) or clinical pharmacy (20.2%). A large proportion of the participants with a master’s degree were in majors of other pharmacy (21.1%), such as pharmacology, pharmacoanalysis, etc.

**Table 2:** Education Background of Clinical Pharmacists

Item	N (%)
Highest education	
Lower than bachelor’s degree	33 (5.3)
Bachelor’s degree	334 (53.4)
Master’s degree	247 (39.5)
Doctoral degree	11 (1.8)
Qualifications of practice	
National-level specialized training	253 (40.5)
National-level general training	128 (20.5)
Province-level specialized training	130 (20.8)
Province-level general training	97 (15.5)
Training experience	
Certificate of completion of clinical pharmacist training from the Ministry of Health	334 (53.4)
Advanced training certificate of clinical pharmacist	190 (30.4)
Certificate of training faculty of clinical pharmacist of National Health and Family Planning Commission	149 (23.8)
Overseas training for clinical pharmacist	16 (2.6)
Other training	88 (14.1)
Type of lower than bachelor’s degree	
None	570 (91.2)
Pharmacy	31 (5.0)
Clinical pharmacy	7 (1.1)
Chinese materia medica	4 (0.6)
Other pharmacy	3 (0.5)
Clinical medicine	0 (0.0)
Other medical specialties	0 (0.0)
Other	10 (1.6)
Type of bachelor’s degree	
None	75 (12.0)
Pharmacy	296 (47.4)
Clinical pharmacy	126 (20.2)
Chinese materia medica	26 (4.2)
Other pharmacy	70 (11.2)
Clinical medicine	24 (3.8)
Other medical specialties	3 (0.5)
Other	5 (0.8)
Type of master’s degree	
None	367 (58.7)
Pharmacy	53 (8.5%)
Clinical pharmacy	49 (7.8)
Chinese materia medica	19 (3.0)
Other pharmacy	132 (21.1)
Clinical medicine	4 (0.6)
Other medical specialties	1 (0.2)
Other	0 (0.0)
Type of doctoral degree	
None	614 (98.2)
Pharmacy	1 (0.2)
Clinical pharmacy	4 (0.6)
Chinese materia medica	0 (0.0)
Other pharmacy	6 (1.0)
Clinical medicine	0 (0.0)
Other medical specialties	0 (0.0)
Other	0 (0.0)

^a^Some participants obtained the bachelors-masters degrees, and several participants only provided their highest education. Therefore, the statistical results of highest education are different from those of specific education level in the table

For qualifications of practice, participants who received national-level specialized or general training, provincial-level specialized orgeneral training accounted for 40.5, 20.5, 20.8 and 15.5%, respectively. In addition, 17.4% of the participants have none of the above qualifications.

For training experience, most of the participants obtained certificate of completion of clinical pharmacist training from the Ministry of Health (53.4%)Some of them were with advanced training certificate of clinical pharmacist (30.4%), or certificate of training faculty of clinical pharmacist of National Health and Family Planning Commission (23.8%).

The results of the regression analysis are provided in Table 3. Among participants with less than bachelor’s degrees, those majoring in general pharmacy (coef. = − 2.39, *p* = 0.09, 95%CI = [− 5.11,0.33]) and Chinese materia medica (coef. = − 3.53, *p* = 0.39, 95%CI = [− 11.64,4.58]) had lower scores of the clinical pharmacy workload, whereas those who majoring in other specialties (coef. = 5.40, *p* = 0.01, 95%CI = [1.35,9.45]) had higher scores significantly. Among participants with bachelor’s degrees，those majoring in clinical pharmacy (coef. = 1.18, *p* = 0.30, 95%CI = [− 1.04,3.41]) and other majors (coef. = 1.25, *p* = 0.62, 95%CI = [− 3.67,6.16]) had higher score of the clinical pharmacy workload, whereas those majoring in clinical medicine (coef. = − 0.23, *p* = 0.85, 95%CI = [− 2.60,2.14]) had lower scores. Among participants with master’s degrees，those majoring in Chinese materia medica (coef. = 1.96, *p* = 0.39, 95%CI = [− 2.52,6.44]) and clinical medicine (coef. = 1.30, *p* = 0.39, 95%CI = [− 1.69,4.30]) had higher scores of the clinical pharmacy workload, whereas those majoring in other medical specialties (coef. = − 8.61, *p* = 0.16, 95%CI = [− 20.61,3.39]) had lower scores. Among participants with doctoral degrees，those who majoring in clinical pharmacy (coef. = 7.30, *p* = 0.00, 95%CI = [2.73,11.87]) had highest scores of the clinical pharmacy workload.

**Table 3:** The Regression Result

Item	Original Research			The Result of Robustness Test		
	Coef.	95%CI	***p***-value	Coef.	95%CI	***p***-value
Type of lower than bachelor’s degree (ref = none)						
Pharmacy	−2.39	[−5.11,0.33]	0.09^*^	−2.34	[−4.98,0.30]	0.08^*^
Clinical pharmacy	−0.23	[−3.99,3.52]	0.90	−1.41	[−5.26,2.44]	0.47
Chinese materia medica	−3.53	[−11.64,4.58]	0.39	−3.15	[− 12.56,6.27]	0.53
Other pharmacy	−0.58	[−7.91,6.75]	0.88	−0.64	[−6.77,5.50]	0.84
Clinical medicine	–	–	–	–	–	–
Other medical specialties	–	–	–	–	–	–
Other	5.40	[1.35,9.45]	0.01^***^	4.46	[0.41,8.51]	0.03^**^
Type of bachelor’s degree (ref = none)						
Pharmacy	0.93	[−0.91,2.77]	0.32	0.66	[−1.21,2.54]	0.49
Clinical pharmacy	1.18	[−1.04,3.41]	0.30	1.13	[−1.11,3.37]	0.32
Chinese materia medica	0.16	[−5.57,4.89]	0.95	0.32	[−4.06,4.70]	0.89
Other pharmacy	−0.08	[−2.36,2.20]	0.95	0.08	[−2.32,2.40]	0.94
Clinical medicine	−0.23	[−2.60,2.14]	0.85	−0.09	[−2.51,2.33]	0.94
Other medical specialties	0.55	[−11.31,12.41]	0.93	−0.08	[−9.7,9.55]	0.99
Other	1.35	[−3.67,6.16]	0.62	1.68	[−2.77,6.13]	0.46
Type of master’s degree (ref = none)						
Pharmacy	−1.10	[−2.69,0.48]	0.17	−1.14	[−2.76,0.47]	0.16
Clinical pharmacy	0.45	[−1.59,2.49]	0.67	0.31	[−1.55,2.17]	0.74
Chinese materia medica	1.96	[−2.52,6.44]	0.39	1.70	[−2.47,5.87]	0.42
Other pharmacy	0.38	[−1.10,1.87]	0.61	0.16	[−1.28,1.60]	0.82
Clinical medicine	1.30	[−1.69,4.30]	0.39	1.59	[−0.98,4.16]	0.23
Other medical specialties	−8.61	[−20.61,3.39]	0.16	−6.82	[−16.58,2.95]	0.17
Other	–	–	–	–	–	–
Type of doctoral degree (ref = none)						
Pharmacy	1.17	[−2.57,4.91]	0.54	1.98	[−1.82,5.78]	0.31
Clinical pharmacy	7.30	[2.73,11.87]	0.00^***^	6.30	[2.09,10.51]	0.00^***^
Chinese materia medica	–	–	–	–	–	–
Other pharmacy	1.95	[−4.83,8.72]	0.57	2.35	[−4.19,8.90]	0.48
Clinical medicine	–	–	–	–	–	–
Other medical specialties	–	–	–	–	–	–
Other	–	–	–	–	–	–
Qualifications of practice (ref = not obtained)						
National-level specialized training	2.42	[1.04,3.80]	0.00^***^	2.56	[1.25,3.87]	0.00^***^
National-level general training	0.35	[−1.08,1.78]	0.63	0.34	[−1.01,1.69]	0.62
Province-level specialized training	1.19	[−0.17,2.54]	0.09^*^	1.33	[0.06,2.60]	0.04^*^
Province-level general training	0.84	[−0.72,2.39]	0.29	1.24	[−0.26,2.74]	0.10
Training experience(ref = not acquired)						
Certificate of completion of clinical pharmacist training from the Ministry of Health	0.03	[−1.51,1.58]	0.96	−0.27	[− 1.75,1.21]	0.72
Advanced training certificate of clinical pharmacist	−0.24	[−1.80,1.33]	0.77	−0.38	[−1.89,1.12]	0.62
Certificate of training faculty of clinical pharmacist of National Health and Family Planning Commission	0.64	[−0.89,2.16]	0.41	0.19	[−1.25,1.64]	0.79
Overseas training for clinical pharmacist	1.67	[−1.80,5.14]	0.34	1.29	[−2.27,4.85]	0.48
Other training	−0.87	[−3.01,1.26]	0.42	−1.12	[−3.16,0.92]	0.28
Gender(ref = male)						
Female	−0.35	[−1.45,0.75]	0.54	−0.34	[−1.41,0.73]	0.53
Age	−0.04	[−0.18,0.10]	0.60	−0.03	[− 0.17,0.10]	0.66
Marital status(ref = unmarried)						
Married	−0.27	[−1.86,1.33]	0.74	−0.33	[−1.83,1.17]	0.67
Other (divorced, window, etc.)	−1.00	[−8.12,6.11]	0.78	−0.21	[−6.64,6.21]	0.95
Years of practice	0.04	[−0.09,0.16]	0.56	0.02	[−0.1,0.13]	0.80
Technical titles (ref = junior title)						
Intermediate title	0.35	[−1.00,1.70]	0.61	0.48	[−0.85,1.81]	0.48
Deputy senior title	1.03	[−0.99,3.05]	0.32	1.28	[−0.68,3.25]	0.20
Senior title	0.47	[−4.25,5.20]	0.84	1.03	[−3.83,5.88]	0.68
Specialized field (ref = unengaged)						
Anti-infectives	0.85	[−0.45,2.15]	0.20	0.77	[−0.50,2.03]	0.23
Cardiology	−0.01	[−1.57,1.56]	0.99	−0.17	[− 1.69,1.34]	0.82
Respiratory medicine	1.63	[0.08,3.17]	0.04^**^	1.37	[−0.06,2.81]	0.06^*^
Gastroenterology	1.35	[−0.24,2.94]	0.10^*^	0.87	[−0.68,2.42]	0.27
Nephrology	3.10	[1.13,5.07]	0.00^***^	3.30	[1.59,5.01]	0.00^***^
Oncology	0.16	[−1.4,1.72]	0.84	0.15	[−1.37,1.68]	0.84
Organ Transplantation	−1.86	[−5.57,1.85]	0.33	−1.4	[−5.67,2.88]	0.52
ICU	0.77	[−0.97,2.50]	0.39	0.92	[− 0.84,2.68]	0.30
Endocrinology	−0.15	[−2.12,1.82]	0.88	0.33	[−1.59,2.25]	0.74
Neurology	1.12	[−0.80,3.03]	0.25	1.08	[−0.70,2.87]	0.23
Other	0.58	[−0.99,2.16]	0.47	0.01	[−1.58,1.60]	0.99
Type of hospital (ref = general hospital)						
Specialized hospital	−1.90	[−4.93,1.13]	0.22	−1.48	[−4.43,1.46]	0.32
Traditional Chinese medicine hospital	−0.52	[−1.88,0.84]	0.45	−0.69	[−2.03,0.66]	0.32
Other	−1.19	[−4.67,2.29]	0.45	−0.69	[−2.03,0.66]	0.32
Constant	43.10	[38.47,47.72]	0.00^***^	45.62	[41.16,50.08]	0.00^***^
I am satisfied with my job (ref = agree)						
somewhat agree				−2.95	[−3.97,-1.92]	0.00^***^
somewhat disagree				−5.93	[−8.82,-3.05]	0.00^***^
disagree				1.17	[− 3.73,6.08]	0.64

^*^p < .1, ^**^p < .05, ^***^p < .01

The participants who obtained national-level (coef. = 2.42, *p* = 0.00, 95%CI = [1.04,3.80]) or provincial-level(coef. = 1.19, *p* = 0.09, 95%CI = [− 0.17,2.54]) specialized training had higher scores of the clinical pharmacy workload than those obtained national-level (coef. = 0.35, *p* = 0.63, 95%CI = [− 1.08,1.78]) or provincial-level (coef. = 0.84, *p* = 0.29, 95%CI = [− 0.72,2.39]) general training. Clinical pharmacists who had participated in overseas training (coef. =1.67, *p* = 0.34, 95%CI = [− 1.80,5.14]) had high scores of the clinical pharmacy workload.

## Discussion

This study focused on the relationship of education background of clinical pharmacists in tertiary hospitals in China and their clinical pharmacy workload. The sample has a similar distribution of gender, age and technical titles to those indicators of the sample reported in a national study of clinical pharmacists in China [20], indicating acceptable representativeness of the sample. The results revealed that clinical pharmacists with less than bachelor’s degree in general pharmacy, doctoral degree in clinical pharmacy, national-level and provincial-level specialized training, and the type of specialized field they engaged in, such as respiratory medicine and nephrology, were associated with their clinical workload significantly.

The results revealed that participants with doctoral degrees in clinical pharmacy have a significantly high degree of workload completion. This is consistent with the findings of the study on pharmacy technicians [44]. The reason may be that educational level reflects the professional level to some extent, clinical pharmacists with doctoral degrees having more knowledge and experience. Compared with doctoral degrees in other pharmacy-related specialties, the training objectives of clinical pharmacy are more suitable for the responsibilities of clinical pharmacists. The results of this study support the view that clinical pharmacists with a doctoral degree in clinical pharmacy are more in line with the functions of tertiary hospitals in China to treat critical disease, which could provide a reference for the training direction of clinical pharmacists in tertiary hospitals in China.

The regression results for clinical pharmacists with less than a bachelor’s degree seem to be counterintuitive. This can be understood in terms of clinical experience rather than educational background. In China clinical pharmacists have been required to have a bachelor’s degree or higher since 2011, while in-service clinical pharmacists with substandard education were allowed to continue practicing after standardized training. For this historical reason, a small number of clinical pharmacists with substandard educational backgrounds have rich clinical experience and are capable of completing clinical pharmacy work.

Compared with those received general training, the clinical pharmacists who received national-level and provincial-level specialized training can complete significantly high degree of pharmacy workload, indicating that specialized training meets the practical needs of clinical pharmacists and is more conducive to improving their professional proficiency [22]. This may be related to the training content and mode. Specialized training is subject-specific and involves in-depth professional knowledge, so it is more suitable for the positioning of treating critical disease of tertiary hospitals. Whereas the general training just involves basic knowledge and skills of pharmaceutical care, which covers a wide range of knowledge but the degree of specialization is relatively low. It would be more suitable for the clinical pharmacists working in primary or secondary medical institutions to provide general healthcare. The training of clinical pharmacists is a bridge linking higher education and clinical practice, for it can help the pharmacists to apply their theoretical knowledge expertly. In-depth specialized training may be an important way to improve the clinical pharmacy workload for tertiary hospitals in China.

The training objectives of clinical pharmacy education accord with the work requirements of clinical pharmacists. But the results revealed that whether majoring in clinical pharmacy had no significant association with the clinical pharmacy workload in this sample of clinical pharmacists with a bachelor’s or master’s degree. This could be because the undergraduate education of clinical pharmacy has been emphasizing theory over practice in China [45], and the curricular follows the characteristic of drug-centered [46], which results in a lack of clinical treatment skills for students. And clinical pharmacy graduate education focuses on scientific research rather than capacity for clinical drug administration or pharmaceutical care skills, which is not conductive for students of this major to be competent in a patient-centered work mode [47]. Therefore, it is recommended to strengthen training their drug treatment-related professional skills to improve their clinical practice ability Based on needs of clinical pharmaceutical care in China, we could learn from the American experience of setting curriculum around professional knowledge, skills, attitudes and values [48], ultimately cultivating service-oriented clinical pharmacy professionals.

Diverse professional backgrounds are one of the characteristics of clinical pharmacist workforce in China which may result from some dilemmas in this field, such as shortage of clinical pharmacy professionals [49, 50] or the need for integrating into clinical treatment teams rapidly [51]. The principal contradiction is that the knowledge structure varies greatly among different majors [52]. Compared with the national standards for undergraduate education in clinical pharmacy, medical graduates lack drug-related knowledge, and pharmacy (non-clinical pharmacy) graduates lack the skills to participate in clinical practice. Lack of drug and clinical knowledge and skills is not conducive to conducting clinical pharmacy work. Therefore, the in-service education and continuing education of clinical pharmacists could pay more attention to the training of clinical pharmacy knowledge and skills.

Clinical pharmacists working in respiratory medicine and nephrology undertook significantly high clinical pharmacy workload, whereas those working in organ transplantation undertook low clinical pharmacy workload. This maybe because clinical problems of respiratory medicine and nephrology are common which have corresponding processing experience in everyday work thus the application of clinical skills in these departments are mature. Whereas individual patients vary greatly in department of organ transplantation, so the irclinical pharmaceutical care and medication is complex and highly demanded, which makes it difficult for clinical pharmacists to be competent. The regression results suggest that the training duration and depth of organ transplantation, endocrinology and cardiology could be appropriately expanded.

This study has some limitations. First, the convenience sampling used in the stratified sampling procedure may have led to a biased sample; however, the socio-demographics of the sample was relatively consistent with the socio-demographics of clinical pharmacists from tertiary hospitals reported in a previous study. Second, the SPCP was originally in English, and the version used in the study was translated from English to Chinese. Although the translated version was determined to have acceptable reliability within this sample, there could still be limitations associated with the use of this translated instrument before being tested among a larger sample with formal validation analyses.

## Conclusion

This study analyzed the association of education background and the clinical pharmacy workload of clinical pharmacists in tertiary hospitals of China through a nationwide survey. The results showed that clinical pharmacists with less than bachelor’s degree in general pharmacy, doctoral degree in clinical pharmacy, national-level and province-level specialized training, and types of specialized field they engaged in, such as respiratory medicine and nephrology, were significantly associated with their clinical pharmacy workload. These results may provide policy makers, the higher education system, clinical pharmacist training organizations, and hospital administrators with the evidence to support the improvement of clinical pharmacy training in China and possibly other developing countries.

## Supplementary Information

**Additional file 1: Appendix 1.** Questionnaire for education background and workload of clinical pharmacists (English version).

**Additional file 2: Appendix 2 Table 1.** The score of clinical pharmacists’ workload.

## Data Availability

The datasets generated during the current study are not publicly available because that are being used for other ongoing researches. But the datasets are available on reasonable request by contacting the corresponding author Xiaoyu Xi.

## Footnotes

1 The coverage rate of clinical responsibilities reflects the number of clinical pharmacy services.
