# Supplementary material for: Association of education background with clinical pharmacists’ clinical pharmacy workload in tertiary hospitals of China
Source: BMC Med Educ. 2022 Nov 9;22:769. doi: 10.1186/s12909-022-03859-w (PMC9648015; doi:10.1186/s12909-022-03859-w)
Supplement: Supplementary file 1 — Additional file 1: Appendix 1. Questionnaire for education background and workload of clinical pharmacists (English version). [file 12909_2022_3859_MOESM1_ESM.docx]

Appendix 1: Questionnaire

Questionnaire for education background and workload of clinical pharmacists (English version)

| Dear clinical pharmacist:  This is a questionnaire about the education background and workload of clinical pharmacists in China. It aims to study the impact of education background on the clinical pharmacy workload of clinical pharmacists, so as to provide information and suggestions for the development of the education model of clinical pharmacists in China. The survey results do not contain any of your identity information, and the survey results will be kept strictly confidential and used only for academic research and not for any commercial purpose.  The questions in the questionnaire are all single-choice questions or blank questions. Please answer according to your actual situation and attitude. If you have any questions about the requirements of the questionnaire, please feel free to ask the investigator. Thank you for your support and cooperation. | |
| --- | --- |
| Section 1 | |
| 1. What is your gender? | □Male □Female |
| 2. What is your age? | _____years old |
| 3. What is your current marriage situation? | □Unmarried □Married □Other (Divorced, Widowed, etc.) |
| 4. How many years have you worked in your current or similar position? | _____years |
| 5. What is your technical title? | □Junior title □Intermediate title □Deputy senior title □senior title |
| 6. What is your degree and major at each level? (Multiple choices) | □Lower than Bachelor degree, major_____ □Bachelor degree, major_____ □Master degree, major_____ □Doctoral degree, major_____ |
| 7. What qualifications do you have for clinical pharmacists? (Multiple choices) | □Nation-level Specialized Training □Nation-level General Training □Province-level Specialized Training □Province-level General Training |
| 8. What is the name of your current profession? (Multiple choices) | □Anti-infectives □Cardiology □Respiratory Medicine □Gastroenterology □Nephrology □Oncology □Organ transplantation □Intensive care □Endocrinology □Neurology □Other |
| 9. What is the type of the hospital you work in? | □ General hospital □Specialized hospital  □Traditional Chinese Medicine Hospital □Other |
| 10. What training experience and qualifications do you have for clinical pharmacists? (Multiple choices) | □Certificate of completion of clinical pharmacist training from the Ministry of Health □Advanced training certificate of clinical pharmacist □Certificate of training faculty of clinical pharmacist of National Health and Family Planning Commission □Overseas training for clinical pharmacist □Other training |

| 11. you are taking the following duties: | |
| --- | --- |
| (1) Assessing the requirements of patients’ medication | □ Strongly disagree □Disagree □Uncertain □Agree □Strongly agree |
| (2) Evaluating the rationality, safety, efficacy, economy, patients ‘compliance and potential problems of medication | □ Strongly disagree □Disagree □Uncertain □Agree □Strongly agree |
| (3) Formulating and implementing health care plan | □ Strongly disagree □Disagree □Uncertain □Agree □Strongly agree |
| (4) Conducting follow-up evaluation and drug monitoring | □ Strongly disagree □Disagree □Uncertain □Agree □Strongly agree |
| (5) Recording information on patients’ mediation | □ Strongly disagree □Disagree □Uncertain □Agree □Strongly agree |
| (6) Checking medication history | □ Strongly disagree □Disagree □Uncertain □Agree □Strongly agree |
| (7) Summarizing and assessing the problems of mediation | □ Strongly disagree □Disagree □Uncertain □Agree □Strongly agree |
| (8) Optimizing mediation and improve the prognosis of patients | □ Strongly disagree □Disagree □Uncertain □Agree □Strongly agree |
| (9) Collaborating with other health care providers in the medical team and performing your own unique responsibilities | □ Strongly disagree □Disagree □Uncertain □Agree □Strongly agree |
| (10) Maintaining and improving professional competence | □ Strongly disagree □Disagree □Uncertain □Agree □Strongly agree |

| (11) Scientific research and academic work | □ Strongly disagree □Disagree □Uncertain □Agree □Strongly agree |
| --- | --- |

| (12) Teaching and guidance | □ Strongly disagree □Disagree □Uncertain □Agree □Strongly agree |
| --- | --- |
