# Supplementary material for: Association of education background with clinical pharmacists’ clinical pharmacy workload in tertiary hospitals of China
Source: BMC Med Educ. 2022 Nov 9;22:769. doi: 10.1186/s12909-022-03859-w (PMC9648015; doi:10.1186/s12909-022-03859-w)
Supplement: Supplementary file 2 — Additional file 2: Appendix 2 Table 1. The score of clinical pharmacists’ workload. [file 12909_2022_3859_MOESM2_ESM.docx]

Appendix 2

Table 1 The score of clinical pharmacists’ workload

| **Item** | **Strongly disagree (N, %)** | **Disagree (N, %)** | **Uncertain (N, %)** | **Agree (N, %)** | **Strongly agree (N, %)** | **Mean (SD)** |
| --- | --- | --- | --- | --- | --- | --- |
| (1) Assessing the requirements of patients’ medication | 7(0.5%) | 120(9.2%) | 160(12.3%) | 883(67.9%) | 130(10%) | 3.78(0.77) |
| (2) Evaluating the rationality, safety, efficacy, economy, patients ‘compliance and potential problems of medication | 5(0.4%) | 83(6.4%) | 116(8.9%) | 925(71.2%) | 171(13.2%) | 3.90(0.71) |
| (3) Formulating and implementing health care plan | 26(2.0%) | 299(23.0%) | 318(24.5%) | 570(43.8%) | 87(6.7%) | 3.30(0.96) |
| (4) Conducting follow-up evaluation and drug monitoring | 9(0.7%) | 146(11.2%) | 178(13.7%) | 840(64.6%) | 127(9.8%) | 3.72(0.82) |
| (5) Recording information on patients’ mediation | 24(1.8%) | 231(17.8%) | 181(13.9%) | 732(56.3%) | 132(10.2%) | 3.55(0.96) |
| (6) Checking medication history | 8(0.6%) | 105(8.1%) | 130(10.0%) | 891(68.5%) | 166(12.8%) | 3.85(0.76) |
| (7) Summarizing and assessing the problems of mediation | 3(0.2%) | 81(6.2%) | 116(8.9%) | 931(71.6%) | 169(13.0%) | 3.91(0.69) |
| (8) Optimizing mediation and improve the prognosis of patients | 12(0.9%) | 185(14.2%) | 232(17.8%) | 757(58.2%) | 114(8.8%) | 3.60(0.87) |
| (9) Collaborating with other health care providers in the medical team and performing your own unique responsibilities | 6(0.5%) | 77(5.9%) | 152(11.7%) | 898(69.1%) | 167(12.8%) | 3.88(0.71) |
| (10) Maintaining and improving professional competence | 4(0.3%) | 41(3.2%) | 90(6.9%) | 962(74.0%) | 203(15.6%) | 4.01(0.62) |
| (11) Scientific research and academic work | 32(2.5%) | 274(21.1%) | 232(17.8%) | 641(49.3%) | 121(9.3%) | 3.42(1.00) |
| (12) Teaching and guidance | 38(2.9%) | 290(22.3%) | 203(15.6%) | 636(48.9%) | 133(10.2%) | 3.41(1.03) |
| Total | - | - | - | - | - | 44.33(4.65) |
